# Visualizing genome and systems biology: technologies, tools, implementation techniques and trends, past, present and future

**DOI:** 10.1186/s13742-015-0077-2

**Published:** 2015-08-25

**Authors:** Georgios A. Pavlopoulos, Dimitris Malliarakis, Nikolas Papanikolaou, Theodosis Theodosiou, Anton J. Enright, Ioannis Iliopoulos

**Affiliations:** 1 Bioinformatics & Computational Biology Laboratory, Division of Basic Sciences, University of Crete, Medical School, 70013 Heraklion, Crete Greece; 2 Department of Biology, University of Crete, 70013 Heraklion, Crete Greece; 3 EMBL - European Bioinformatics Institute, Wellcome Trust Genome Campus, Cambridge, CB10 1SD UK

**Keywords:** Biological data visualization, Network biology, Genomics, Systems biology, Multivariate analysis

## Abstract

“Α picture is worth a thousand words.” This widely used adage sums up in a few words the notion that a successful visual representation of a concept should enable easy and rapid absorption of large amounts of information. Although, in general, the notion of capturing complex ideas using images is very appealing, would 1000 words be enough to describe the unknown in a research field such as the life sciences? Life sciences is one of the biggest generators of enormous datasets, mainly as a result of recent and rapid technological advances; their complexity can make these datasets incomprehensible without effective visualization methods. Here we discuss the past, present and future of genomic and systems biology visualization. We briefly comment on many visualization and analysis tools and the purposes that they serve. We focus on the latest libraries and programming languages that enable more effective, efficient and faster approaches for visualizing biological concepts, and also comment on the future human-computer interaction trends that would enable for enhancing visualization further.

## Review

### Introduction

In the current ‘big data’ era [1], the magnitude of data explosion in life science research is undeniable. The biomedical literature currently includes about 27 million abstracts in PubMed and about 3.5 million full text articles in PubMed Central. Additionally, there are more than 300 established biological databases that store information about various biological entities (bioentities) and their associations. Obvious examples include: diseases, proteins, genes, chemicals, pathways, small molecules, ontologies, sequences, structures and expression data. In the past 250 years, only 1.2 million eukaryotic species (out of the approximately 8.8 million that are estimated to be present on earth) [2] have been identified and taxonomically classified in the Catalog of Life and the World Register of Marine Species [3]. The sequencing of the first human genome (2002) took 13 years and cost over $3 million to complete. Although the cost for *de novo* assembly of a new genome to acceptable coverage is still high, probably at least $40,000, we can now resequence a human genome for $1000 and can generate more than 320 genomes per week [4]. Notably, few species have been fully sequenced, and a large fraction of their gene function is not fully understood or still remains completely unknown [5]. The human genome is 3.3 billion base pairs in length and consists of over 20,000 human coding genes organized into 23 pairs of chromosomes [6, 7]. Today over 60,000 solved protein structures are hosted in the Protein Data Bank [8]. Nevertheless, many of the protein functions remain unknown or are partially understood.

Shifting away from basic research to applied sciences, personalized medicine is on the cusp of a revolution allowing the customization of healthcare by tailoring decisions, practices and/or products to the individual patient. To this end, such information should be accompanied by medical history and digital images and should guarantee a high level of privacy. The efficiency and security of distributed cloud computing systems for medical health record organization, storage and handling will be one of the big challenges during the coming years.

Information overload, data interconnectivity, high dimensionality of data and pattern extraction also pose major hurdles. Visualization is one way of coping with such data complexity. Implementation of efficient visualization technologies is necessary not only to present the known but to also reveal the unknown, allowing inference of conclusions, ideas and concepts [9]. Here we focus on visualization advances in the fields of network and systems biology, present the state-of-the-art tools and provide an overview of the technological advances over time, gaining insights into what to expect in the future of visualization in the life sciences.

In the section on network biology below, we discuss widely used tools related to graph visualization and analysis, we comment on the various network types that often appear in the field of biology and we summarize the strengths of the tools, along with their citation trends over time. In this section we also distinguish between tools for network analysis and tools designed for pathway analysis and visualization. In a section on genomic visualization, we follow the same approach by distinguishing between tools designed for genome browsing and visualization, genome assembly, genome alignments and genome comparisons. Finally, in a section on visualization and analysis of expression data, we distinguish between tree viewers and tools implemented for multivariate analysis.

### Network biology visualization

**Fig. 1:**
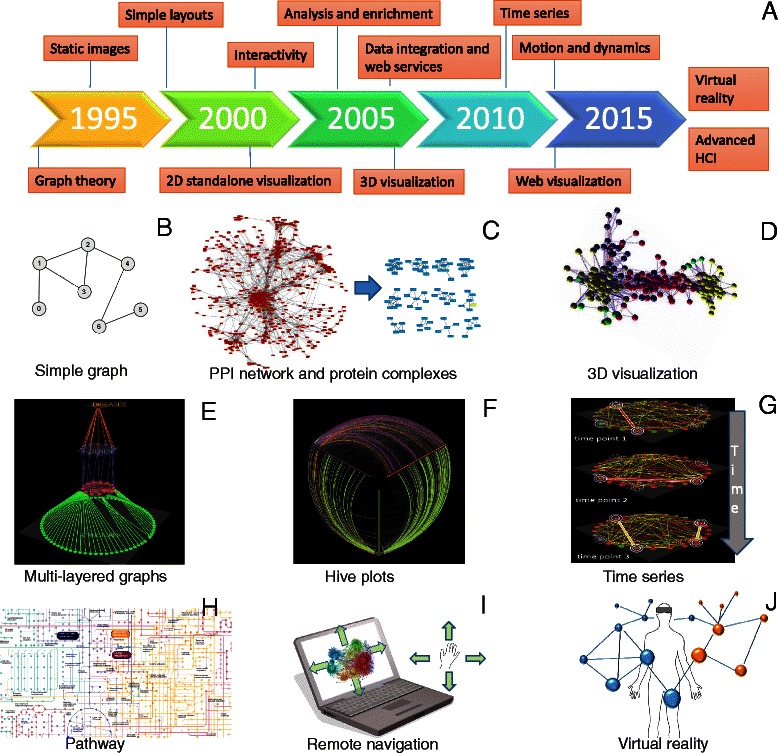
Visualization for network biology. **a** Timeline of the emergence of relevant technologies and concepts. **b** A simple drawing of an undirected unweighted graph. **c** A 2D representation of a yeast protein-protein interaction network visualized in Cytoscape (*left*) and potential protein complexes identified by the MCL algorithm from that network (*right*). **d** A 3D view of a protein-protein interaction network visualized by BiolayoutExpress^3D^. **e** A multilayered network integrating different types of data visualized by Arena3D. **f** A hive plot view of a network in which nodes are mapped to and positioned on radially distributed linear axes. **g** Visualization of network changes over time. **h** Part of lung cancer pathway visualized by iPath. **i** Remote navigation and control of networks by hand gestures. **j** Integration and control of 3D networks using VR devices

In the field of systems biology, we often meet network representations in which bioentities are interconnected with each other. In such graphs, each node represents a bioentity and edges (connections) represent the associations between them [10]. These graphs can be weighted, unweighted, directed or undirected. Among the various networks types within the field, some of the most widely used are protein-protein interaction networks, literature-based co-occurrence networks, metabolic/biochemical, signal transduction, gene regulatory and gene co-expression networks [11–13]. As new technological advances and high-throughput techniques come to the forefront every few years, such networks can increase dramatically in size and complexity, and therefore more efficient algorithms for analysis and visualization are necessary. Notably, a network consisting of a hundred nodes and connections is incomprehensible and impossible for a human to visually analyze. For example, techniques such as tandem affinity purification (TAP) [14], yeast two hybrid (Y2H) [15] and mass spectrometry [16] can nowadays generate a significant fraction of the physical interactions of a proteome. As network biology evolves over time, we indicate standard procedures that were developed over the past 20 years and highlight key tools and methodologies that had a crucial role in this maturation process (Fig. 1).

In the 1990s, two-dimensional (2D) static graph layouts were developed for visualizing networks. Topological analysis, layout and clustering were pre-calculated and results were captured in a single static image. Clustering analysis was performed to detect cliques or highly connected regions within a graph, layout techniques such as Fruchterman-Reingold [17] were implemented to place nodes in positions where the crossovers between the edges are minimized and topological analysis was used for detecting important nodes of the network such as hubs or nodes with high betweenness centrality. The typical visual encoding consisted of using arrows for directed graphs, adjusting the thickness of an edge to show the importance of a connection, using the same color for nodes that belong to the same cluster or modifying the node’s size to show its topological features, such as its neighbor connectivity. As integrative biology and high-throughput techniques advanced over the years, the necessity to move away from static images and add interactivity and navigation for easier data exploration became clearer.

Bridging between analysis and visualization became necessary, and tools that incorporated both increased the standards in the field. In clustering analysis, for example, new computational methods such as MCL [18] and variations [19], Cfinder [20], MCODE [21], Clique [22] and others were applied to biological networks to find highly connected regions of importance. DECAFF [23], SWEMODE [24] or STM [25], for example, were developed to predict protein complexes [26] incorporating graph annotations, whereas others such as DMSP [27], GFA [28] and MATISSE [29] were focused on gene-expression data. Most of these algorithms were command-line-based and only few tools such as jClust [30], GIBA [31], ClusterMaker [32] or NeAT [33] have been developed to integrate data in visual environments. These aforementioned techniques along with others are thoroughly discussed elsewhere [26, 34–36].

Although most network visualization tools are standalone applications, they guarantee efficient data exploration and the manipulation of visualization with mouse-hovering supporting actions. Such tools are for example the Pajek [37], Osprey [38], VisANT [39] and others. Next-generation visualization tools took advantage of standard file formats such as BioPAX [40, 41], SBML [42], PSI-MI [43] and CellML [44]; modern, more sophisticated layouts such as Hive-Plots [45]; and the available web services and data integration techniques to directly retrieve and handle information from public repositories on the fly. Functional enrichment of genes using the Gene Ontology (GO) repository [46] is a typical example. Among others, current state-of-the-art tools are the Ondex [47], Cytoscape [48] or Gephi [49], while tools such as iPath [50], PATIKA [51], PathVisio [52] and others [53] are pathway specific.

As biological networks became larger over time, consisting of thousands of nodes and connections, the so-called ‘hairball’ effect, where many nodes are densely connected with each other became very difficult to cope with. A partial solution to this was to shift from 2D representations to three-dimensional (3D) representations. Tools such as Arena3D [54, 55] or BioLayout Express 3D [56] take advantage of 3D space to show data in a virtual 3D universe. BioLayout Express uses whole 3D space to visualize networks, whereas Arena3D implements a multilayered concept to present 2D networks in a stack. Although a 2D network allows immediate visual feedback, a 3D rendering usually requires the user to interact more with the data in a more explorative mode, but can help reveal interesting features potentially hidden in a 2D representation. Although it is debatable whether 3D rendering is better than 2D visualization, hardware acceleration and performance still need to be taken into account when planning 3D visualizations (Fig. 1).

**Fig. 2:**
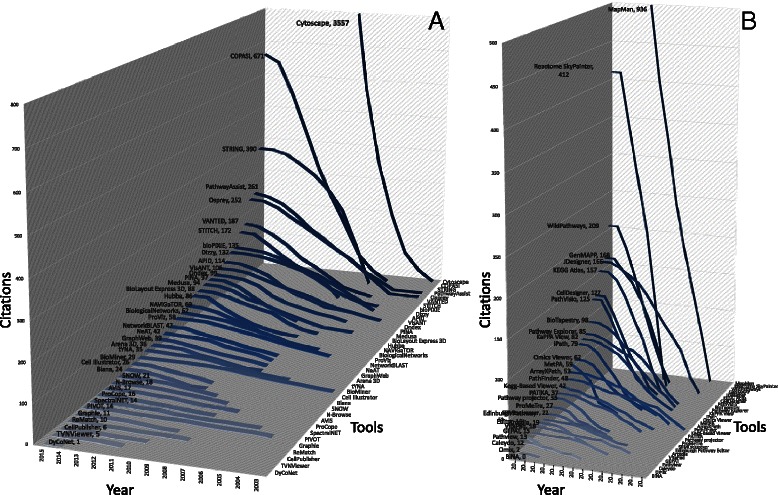
Citation trends and key player tools in network biology. **a** Citations of network visualization tools based on Scopus. **b** Citations of pathway visualization tools based on Scopus. The numbers of citations of each tool in 2015 are shown after its name

Tables 1 and 2 present currently freely available network and pathway visualization tools and their main characteristics. However, it is not the purpose of this review to perform a deeper comparative analysis of all available 2D and 3D visualization tools, as this is available elsewhere [53, 57–59]. Nevertheless, as network biology is gaining ground over the years, we sought to investigate the impact of the current tools in the field. To accomplish this, we tracked the tools that appeared after year 2000 and whose respective articles are indexed by Scopus (Fig. 2). We chose to keep track of the citations of only the first original publication for each tool. Although the number of citations is a reasonable indicator of popularity, it can sometimes be misleading as several tool versions appear in different articles that we have not yet tracked. Nevertheless, some immediate conclusions can be reached, such as that Cytoscape seems to be by far the biggest player for network visualization, as it comes with more than 200 plugins [60] implemented by an active module community (Fig. 1b). Similarly, MapMan [61] and Reactome SkyPainter [62] are the most used tools for pathway visualization (Fig. 2b).

**Table 1:** Visualization tools for network biology

Standalone applications for network analysis		
Tool and references	Description	URL
Arena 3D [54, 55]	3D visualization of multi-layer networks	http://www.arena3d.org
Biana [146]	Data integration and network management	http://sbi.imim.es/web/BIANA.php
BioLayout Express 3D [147]	2D/3D network visualization	http://www.biolayout.org/
BiologicalNetworks [148, 149]	Efficient integrated multi-level analysis of microarray, sequence, regulatory and other data	http://www.biologicalnetworks.org
BioMiner [150]	Modeling, analyzing and visualizing biochemical pathways and networks	http://www.zbi.uni-saarland.de/chair/projects/BioMiner
Cell Illustrator [151]	Petri nets for modeling and simulating biological networks	http://www.cellillustrator.com
COPASI [152]	Analysis of biochemical networks and their dynamics	http://www.copasi.org/
Cytoscape [48, 153]	Network visualization and analysis. Over 200 plugins [60]	http://www.cytoscape.org/
Dizzy [154]	Chemical kinetics stochastic simulation software	http://magnet.systemsbiology.net/software/Dizzy/
DyCoNet [155]	Gephi plugin that can be used to identify dynamic communities in networks	https://github.com/juliemkauffman/DyCoNet
GENeVis [156, 157]	Network and pathway visualization	http://tinyurl.com/genevis/
GEPHI [49]	Interactive visualization and exploration for any network and complex system, dynamic and hierarchical graph.	https://gephi.org
Igraph [158]	Collection of network analysis tools with the emphasis on efficiency, portability and ease of use	http://igraph.sourceforge.net
Medusa [159, 160]	Semantic and multi-edged simple networks	https://sites.google.com/site/medusa3visualization/
NAViGaTOR [161, 162]	Visualizing and analyzing protein-protein interaction networks	http://tinyurl.com/navigator1/
N-Browse [163]	Interactive graphical browser for biological networks	http://www.gnetbrowse.org/
NeAT [33]	Topological and clustering analysis of networks	http://rsat.ulb.ac.be/neat/
Ondex [47]	Data integration and visualization of large networks	http://www.ondex.org/
Osprey [38]	Visualization and annotation of biological networks	http://biodata.mshri.on.ca/osprey/servlet/Index
Pajek [37]	Analysis and visualization of large networks and social network analysis	http://vlado.fmf.uni-lj.si/pub/networks/pajek/
PathwayAssist [164]	Navigation and analysis of biological pathways, gene regulation networks and protein interaction maps.	http://www.ariadnegenomics.com/downloads/
PIVOT [165]	Layout algorithms for visualizing protein interactions and families	http://acgt.cs.tau.ac.il/pivot/
ProCope [166]	Prediction and evaluation of protein complexes from purification data experiments	http://www.bio.ifi.lmu.de/Complexes/ProCope/
ProViz [167]	Visualization and exploration of interaction networks. Gene Ontology and PSI-MI formats supported	http://cbi.labri.fr/eng/proviz.htm
SpectralNET [168]	Network analysis and visualizations. Scatter plots and dimensionality reduction algorithms	https://www.broadinstitute.org/software/spectralnet
Tulip [169]	Enables the development of algorithms, visual encodings, interaction techniques, data models and domain-specific visualizations	http://tulip.labri.fr/TulipDrupal/
VANESA [170]	Automatic reconstruction and analysis of biological networks and Petri nets based on life-science database information	http://agbi.techfak.uni-bielefeld.de/vanesa/
VANTED [171]	Network reconstruction, data visualization, integration of various data types, network simulation	http://tinyurl.com/vanted/
yEd	Creation of diagrams manually and import external data	http://tinyurl.com/yEdGraph/
Web tools for network analysis		
APID [172]	Unified protein-protein interactions from BIND, BioGRID, DIP, HPRD, IntAct and MINT	http://bioinfow.dep.usal.es/apid/
Arcadia [173]	Translates text-based descriptions of biological networks (SBML files) into standardized diagrams (Systems Biology Graphical Notation Process Description maps)	http://arcadiapathways.sourceforge.net/
AVIS [174]	Viewer for signaling networks	http://actin.pharm.mssm.edu/AVIS2
bioPIXIE [175]	Discovery of biological networks from diverse functional genomic data	http://pixie.princeton.edu/pixie
CellPublisher [176]	Interactive representations of biochemical processes	http://cellpublisher.gobics.de/
Graphle [177]	Distributed network exploration and visualization of interactive large, dense graphs	http://tinyurl.com/graphle/
GraphWeb [178]	Web server for graph-based analysis of biological networks	http://biit.cs.ut.ee/graphweb/
Hubba [179]	Web-based service to explore the essential nodes in a network	http://hub.iis.sinica.edu.tw/Hubba
NetworkBLAST [180]	Analysis of protein interaction networks across species to infer protein complexes that are conserved in evolution	http://www.cs.tau.ac.il/~bnet/networkblast.htm
Pathview [181]	Tool set for pathway-based data integration and visualization	http://Pathview.r-forge.r-project.org/
PINA [182]	Integrated platform for protein interaction network construction, filtering, analysis, visualization and management	http://cbg.garvan.unsw.edu.au/pina/home.do
ReMatch [183]	Web-based tool for integration of user-given stoichiometric metabolic models into a database collected from public data sources	http://www.cs.helsinki.fi/group/sysfys/software/rematch/
SNOW [184]	Gene mapping on a reference or human protein-protein interaction network that SNOW hosts	http://snow.bioinfo.cipf.es
STITCH [185]	Resource to explore known and predicted interactions of chemicals and proteins	http://stitch.embl.de/
STRING [186]	Protein interaction networks and integration of data such as genomic context, high-throughput experiments, conserved coexpression and previous knowledge derived from the literature	http://string-db.org
TVNViewer [187]	An interactive visualization tool for exploring networks that change over time or space	http://www.sailing.cs.cmu.edu/main/?page_id=545
tYNA [188]	System for managing, comparing and mining multiple networks	http://tyna.gersteinlab.org/tyna/
VisANT [39, 189]	Visualization, mining, analysis and modeling of biological networks, metabolic networks and ecosystems	http://visant.bu.edu/

**Table 2:** Visualization tools for pathways

Standalone applications		
Tool and references	Description	URL
BiNA [190]	Drawings of metabolic networks supporting hiding of cofactors and drawing of chemical structures	http://bina.unipax.info/
BioTapestry [191]	Interactive tool for building, visualizing and sharing gene regulatory network models over the web	http://www.biotapestry.org/
Caleydo [192]	Visual analysis framework targeted at biomolecular data. Visualization of interdependencies between multiple datasets	http://www.caleydo.org/
CellDesigner [193]	A modeling tool for biochemical networks	http://www.celldesigner.org/
Edinburgh Pathway Editor [194]	Edit and draw pathway diagrams	http://epe.sourceforge.net/SourceForge/EPE.html
GenMAPP [195]	Visualization of gene expression and other genomic data on maps representing biological pathways and groupings of genes	http://www.genmapp.org/
Ingenuity IPA	Data integration platform and manually annotated pathways	http://tinyurl.com/IngenuityPath
JDesigner [196]	Graphical modeling environment for biochemical reaction networks	http://jdesigner.sourceforge.net/Site/JDesigner.html
KaPPA View [197]	Plant pathways	http://kpv.kazusa.or.jp/
KEGG Atlas [198]	Interactive Kyoto Encyclopedia of Genes and Genomes pathways	http://www.genome.jp/kegg/
Omix [199]	Visualizing multi-omics data in metabolic networks	https://www.omix-visualization.com
PathVisio [52]	Biological pathway analysis software that allows drawing, editing and analysis of biological pathways	http://www.pathvisio.org/
VitaPad [200]	Application to visualize biological pathways and map experimental data to them	http://tinyurl.com/vitapad/
Web tools for pathways		
ArrayXPath [201]	Mapping and visualizing microarray gene-expression data and integrated biological pathway resources using SVG	http://tinyurl.com/ArrayXPath/
GEPAT [202]	Integrated analysis of transcriptome data in genomic, proteomic and metabolic contexts	http://gepat.sourceforge.net/
iPath [50, 203]	Web-based tool for the visualization, analysis and customization of pathway maps	http://pathways.embl.de/
Kegg-Based Viewer [204]	KEGG-based pathway visualization tool for complex high-throughput data	http://www.g-language.org/data/marray/
MapMan [61]	User-driven tool that displays large datasets onto diagrams of metabolic pathways or other processes	http://mapman.gabipd.org/web/guest/mapman
MetPA [205]	Analysis and visualization of metabolomic data within the biological context of metabolic pathways	http://metpa.metabolomics.ca
Omics Viewer [206]	Data mapping on BioCyc pathways (collection of 5500 pathway/genome databases)	http://www.biocyc.org/
Pathway Explorer [207]	Interactive Java drawing tool for the construction of biological pathway diagrams in a visual way and the annotation of the components and interactions between them	http://genome.tugraz.at/pathwayexplorer/pathwayexplorer_description.shtml
Pathway projector [208]	Zoomable pathway browser using KEGG atlas and Google Maps API	http://www.g-language.org/PathwayProjector/
PATIKA [51]	Integrated environment composed of a central database and a visual editor, built around an extensive ontology and an integration framework	http://www.cs.bilkent.edu.tr/~patikaweb/
Reactome SkyPainter [62]	Visualization of over-represented pathways and reactions from gene lists	http://www.reactome.org/skypainter-2
WikiPathways [209]	Wiki-based, open, public platform dedicated to the curation of biological pathways by and for the scientific community	http://www.wikipathways.org/

Over the past 5 years, the data visualization field has become more and more competitive. There is a trend away from standalone applications towards the integration of visualization implementations within web browsers. Therefore, libraries and new programming languages have been dedicated to this task (see the final section below). The greater visibility provided by web implementation means that advanced visualization can more easily become available to non-experts and to the broader community. Finally, one of the biggest visualization challenges today is to capture the dynamics of networks and the way in which topological properties change over time [63]. For this, motion or other sophisticated ideas, along with new human-computer interaction (HCI) techniques, should be taken into consideration. Although serious efforts on this are on the way [54, 64, 65], there are still much to expect in the future as HCI techniques and virtual reality (VR) devices (such as Oculus Rift) become cheaper and more advanced over time (Fig. 1).

### Visualization in genomics

**Fig. 3:**
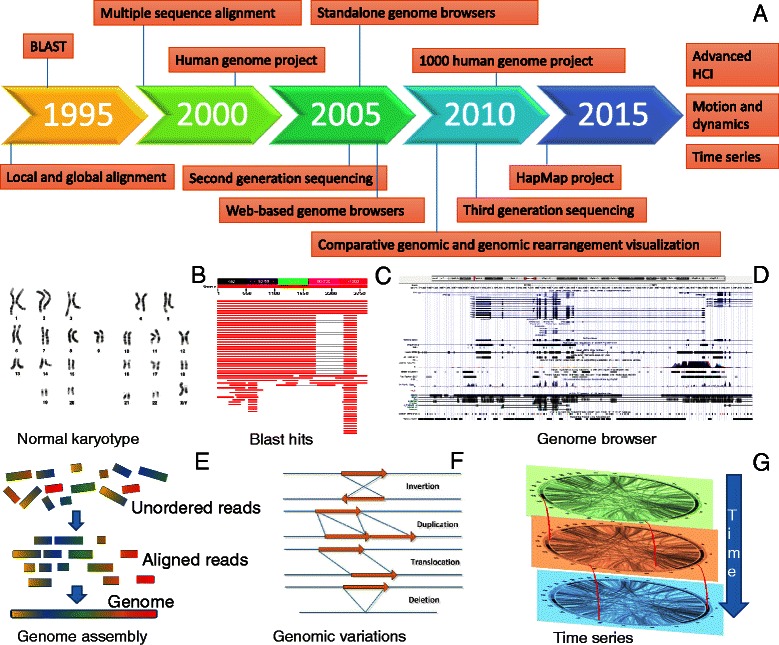
Visualization for genome biology. **a** Timeline of the emergence of relevant technologies and concepts. **b** A typical normal human karyotype. **c** Visualization of BLAST hits and alignment of orthologous genes for the human *TP53* gene. **d** The human *TP53* gene and its annotations visualized by the UCSC genome browser. **e** Visualization of a *de novo* genome assembly from its DNA fragments. **f** Examples of balanced and unbalanced genomic rearrangements. **g** Hypothetical visualization of genomic structural variations across time

There remain many open challenges for advanced visualization for genome assemblies, alignments, polymorphisms, variations, synteny, single nucleotide polymorphisms (SNPs), rearrangements and annotations [66, 67]. To better follow progress in the visualization field, we first need to follow the way in which new technologies, questions and trends have been shaped over the years (Fig. 3).

Up to the 1990s, local and global pairwise and multiple sequence alignment algorithms such as Smith-Waterman [68], Needleman-Wunsch [69], FASTA [70] and BLAST [71] were the focus of bioinformatics methods development. Multiple sequence alignment tools such as the ClustalW/Clustal X [72], MUSCLE [73], T-Coffee [74] and others [75] used basic visualization schemes, in which sequences were represented as strings placed vertically in stacks. Colors were used to visually encode base conservation and to indicate matching, non-matching and similar nucleotides [76, 77].

Although these tools were successful for small numbers of nucleotide or protein sequences, a question was raised regarding their applicability to whole-genome sequencing and comparison. A few years later (2002), the Sanger (dideoxy) first generation sequencing, particularly capillary approaches, allowed the sequencing of the first whole human genome, consisting of about 3 billion base pairs and over 20,000 human genes [78, 79]. Shortly after that, second-generation (Illumina [80], Roche/454 [81], Biosystems/SOLiD [82]) and third-generation techniques (Helicos BioSciences [83], Pacific Biosciences [84], Oxford Nanopore [85] and Complete Genomics [86]) high-throughput sequencing techniques [87–91] allowed the sequencing of a transcriptome, an exome or a whole genome at a much lower cost and within reasonable timeframes.

Projects such as the 1000 Genomes Project, for comprehensive human genetic variation analysis [92–94], and the International HapMap Project [95–99], for the identification of common genetic variations among people from different countries, are just a few examples of the data explosion that has taken place in the era of comparative genomics, after 2005. Such large-scale genomic datasets necessitate powerful tools to link genomic data to its source genome and across genomes. Therefore, among others [66], widely used standalone and web-based genome browsers were dedicated to information handling, genome visualization, navigation, exploration and integration with annotations from various repositories. At present, many specialized tools for comparative genomic visualization are available and are widely used.

**Fig. 4:**
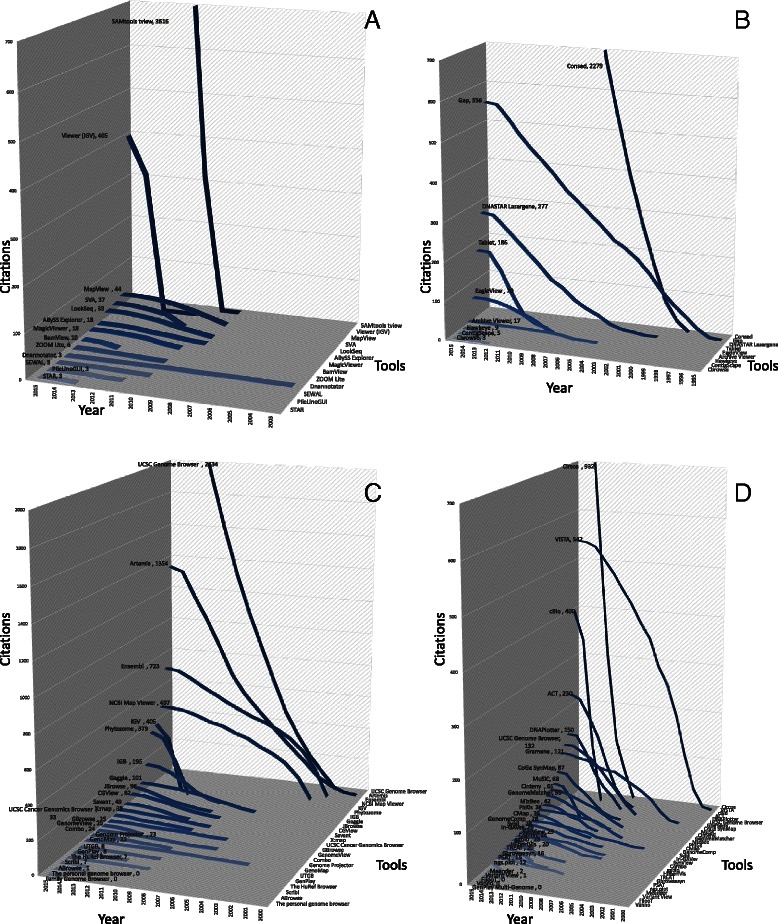
Citation trends and key players in genome biology. **a** Citations of genome alignment visualization tools based on Scopus. **b** Citations of genome assembly visualization tools based on Scopus. **c** Citations of genome browsers based on Scopus. **d** Citations of comparative genomics visualization tools based on Scopus. The numbers of citations of each tool in 2015 are shown after its name

To follow trends in the field, we summarize the tools into four categories: genome alignment visualization tools (Table 3); genome assembly visualization tools (Table 4); genome browsers (Table 5); and tools to directly compare different genomes with each other for efficient detection of SNPs and genomic variations (Table 6). Following the same approach used for network biology (above), we examine the citation progress of the first article that was published for each tool using the Scopus repository (Fig. 4). Consed [76] and Gap [100, 101] seem to be the most widely used assembly viewers, while SAMtools tview [102] is the favorite tool for genomic assembly visualization. In addition, the University of California, Santa Cruz (UCSC) Genome Browser [103], Artemis [104] and Ensembl [105, 106] seem to be the go-to genome browsers, while Circos [107], VISTA [108] and cBio [109] are the most widely used tools for comparative genomics.

**Table 3:** Visualization tools for genome alignments

Tool and references	Description	URL
ABySS Explorer [210]	Interactive Java application that uses a novel graph-based representation to display a sequence assembly and associated metadata	http://www.bcgsc.ca/platform/bioinfo/software/abyss-explorer
BamView [211]	Genome browser and annotation tool that allows visualization of sequence features, next-generation sequencing (NGS) data and the results of analyses within the context of the sequence, and also its six-frame translation	http://www.sanger.ac.uk/resources/software/artemis/
DNannotator [212]	Annotation web toolkit for regional genomic sequences	http://bioapp.psych.uic.edu/DNannotator.htm
JVM [213]	Java Visual Mapping tool for NGS reads	http://www.springer.com/cda/content/document/cda_downloaddocument/9789401792448-c2.pdf?SGWID=0-0-45-1487072-p176815501
LookSeq [214]	Web-based visualization of sequences derived from multiple sequencing technologies. Low- or high-depth read pileups and easy visualization of putative single nucleotide and structural variation	http://lookseq.sourceforge.net
MagicViewer [215]	Visualization of short read alignment, identification of genetic variation and association with annotation information of a reference genome	http://bioinformatics.zj.cn/magicviewer/
MapView [216]	Alignments of huge-scale single-end and pair-end short reads	http://omictools.com/mapview-s1367.html
MultiPipMaker [217]	Computes alignments of similar regions in two DNA sequences. The resulting alignments are summarized with a ‘percent identity plot’ (pip)	http://pipmaker.bx.psu.edu/pipmaker/
PileLineGUI [218]	Handling genome position files in NGS studies	http://sing.ei.uvigo.es/pileline/pilelinegui.html
SAMtools tview [102]	Simple and fast text alignment viewer; NGS compatible	http://www.htslib.org/
SEWAL [219]	Uses a locality-sensitive hashing algorithm to enumerate all unique sequences in an entire Illumina sequencing run	http://www.sourceforge.net/projects/sewal
STAR [220]	A web-based integrated solution to management and visualization of sequencing data	http://wanglab.ucsd.edu/star/browser
SVA [221]	Software for annotating and visualizing sequenced human genomes	http://www.svaproject.org
Viewer (IGV) [222]	Visualization of large heterogeneous datasets, providing a smooth and intuitive user experience at all levels of genome resolution	https://www.broadinstitute.org/igv/
ZOOM Lite [223]	NGS data mapping and visualization software	http://bioinfor.com/zoom/lite/

**Table 4:** Visualization tools for assemblies

Tool and references	Description	URL
Archive Viewer [224]	Web graphical interface to make contigs and trace data changes in the National Center for Biotechnology Information (NCBI)	http://www.ncbi.nlm.nih.gov/Traces/assembly/assmbrowser.cgi?
CBrowse [225]	SAM/BAM-based contig web browser for transcriptome assembly visualization and analysis	http://bioinfolab.muohio.edu/CBrowse/
Consed [76]	Assembly finishing package; NGS compatible	http://www.phrap.org/
ContigScape [226]	A Cytoscape plugin facilitating microbial genome gap closing	http://sourceforge.net/projects/contigscape/
DNASTAR Lasergene [227]	Analysis suite with an assembly package	http://www.dnastar.com/
EagleView [228]	An information-rich viewer for genome assemblies with data integration capability	http://www.niehs.nih.gov/research/resources/software/biostatistics/eagleview/
Gap [100, 101]	A fully developed set of DNA sequence assembly (Gap4 and Gap5), editing and analysis tools	http://staden.sourceforge.net/
Hawkeye [229]	An interactive visual analytics tool for genome assemblies. Detection of anomalies in data and visual identification and correction of assembly errors	http://amos.sourceforge.net/wiki/index.php?title=Hawkeye
Tablet [230]	A lightweight, high-performance graphical viewer for NGS assemblies and alignments	http://bioinf.scri.ac.uk/tablet

**Table 5:** Genome browsers

Tool and references	Description	URL	Web-based or standalone
ABrowse [231]	A customizable genome browser framework	http://www.abrowse.org/	Web-based
AnnoJ [232]	A web 2.0 application designed for visualizing deep sequencing data and other genome annotation data	http://www.annoj.org/	Web-based
Argo	Java tool for visualizing and manually annotating whole genomes	http://www.broadinstitute.org/annotation/argo/	Standalone
Artemis [104]	Browser and annotation tool that allows visualization of sequence features, data and the results of analyses within the context of the sequence, and also its six-frame translation	https://www.sanger.ac.uk/resources/software/artemis/	Standalone
CGView [233]	Static and interactive graphical maps of circular genomes using a circular layout	https://www.gview.ca/wiki/GView/	Standalone
Combo [234]	Dynamic browser to visualize alignments of whole genomes and their associated annotations	http://www.broad.mit.edu/annotation/argo/	Standalone
Ensembl [105, 106]	Annotation, analysis and display of vertebrates and other eukaryotic species	http://www.ensembl.org/	Web-based
Family Genome Browser [235]	Visualizing genomes with pedigree information	http://mlg.hit.edu.cn/FGB/	Web-based
Gaggle [236]	Genome browser within an analysis framework; good microarray support	http://gaggle.systemsbiology.net/	Standalone
GBrowse [237, 238]	A combination of database and interactive web pages for manipulating and displaying annotations on genomes	http://gmod.org/wiki/Gbrowse	Web-based
GenoMap [239]	A circular genome data viewer	http://nsato4.c.u-tokyo.ac.jp/old/GenoMap/GenoMap.html	Standalone
Genome Projector [240]	Circular genome maps, traditional genome maps, plasmid maps, biochemical pathways maps and DNA walks. Google API	http://www.g-language.org/GenomeProjector/	Web-based
GenomeView [241]	Designed to visualize and manipulate a multitude of genomics data	http://genomeview.org/content/integration	Standalone
GenPlay [242]	A multipurpose genome analyzer and browser	http://www.genplay.net	Standalone
IGB [243]	Optimized to achieve maximum flexibility and high quality genome visualization	http://genoviz.sourceforge.net/	Standalone
IGV [222]	A high-performance visualization tool for interactive exploration of large, integrated genomic datasets	http://www.broadinstitute.org/igv/	Standalone
JBrowse [244]	A fast, embeddable genome browser built completely with JavaScript and HTML5	http://jbrowse.org/	Web-based
JGI	Supports live annotation; primary portal for DOE Joint Genomics Institute genome projects	http://genome.jgi-psf.org/	Web-based
NCBI Genome Workbench	An integrated application for viewing and analyzing sequence data	http://www.ncbi.nlm.nih.gov/tools/gbench/	Standalone
NCBI Map Viewer [245]	Vertically oriented viewer; integrated with NCBI resources and tools	http://www.ncbi.nlm.nih.gov/mapview/	Web-based
Phytozome [246]	A comparative platform for green plant genomics	http://www.phytozome.net	Web-based
Savant [247]	It was primarily developed for visualizing sequencing data, although it can be used to visualize almost any genome-based sequence, point, interval or continuous dataset	http://compbio.cs.toronto.edu/savant	Standalone
Scribl [248]	An HTML5 Canvas-based graphics library for visualizing genomic data over the web	http://chmille4.github.com/Scribl/	Web-based
The HuRef Browser [249]	A web resource for individual human genomics	http://huref.jcvi.org	Web-based
The personal genome browser [250]	Visualizing functions of genetic variants	http://www.pgbrowser.org/	Web-based
UCSC Cancer Genomics Browser [251, 252]	Integration of clinical data	http://genome-cancer.ucsc.edu/	Web-based
UCSC Genome Browser [103]	Rapid linear visualization, examination and querying of the data at many levels	http://genome.ucsc.edu/cgi-bin/hgGateway	Web-based
UTGB [253]	Open-source software for developing personalized genome browsers that work in web browsers	http://utgenome.org/	Web-based
X:map [254]	Mappings between genomic features and Affymetrix microarrays	http://xmap.picr.man.ac.uk/	Web-based

**Table 6:** Visualization tools for comparative genomics

Tool and references	Description	URL	Web-based or standalone
ACT [255]	A tool for displaying pairwise comparisons between two or more DNA sequences	http://www.sanger.ac.uk/Software/ACT/	Standalone
cBio [109]	An open-access resource for interactive exploration of multidimensional cancer genomics datasets	http://cbioportal.org	Web-based
Cinteny [256]	Detection of syntenic regions across multiple genomes and measuring the extent of genome rearrangement using reversal distance as a measure	http://cinteny.cchmc.org/	Web-based
Circos [107]	A software package for visualizing data and information. It visualizes data in a circular layout	http://mkweb.bcgsc.ca/circos	Standalone
CMap [257]	A browser-based tool for the visual comparison of various maps (sequence, genetic, etc.) from any number of species	http://gmod.org/wiki/CMap	Standalone
CoGe SynMap [258]	Generates a syntenic dot-plot between two organisms and identifies syntenic regions	https://genomevolution.org/coge/SynMap.pl	Web-based
Combo [234]	Dot-plot and linked-track views. Integration of annotation in both views	http://www.broadinstitute.org/annotation/argo/	Standalone
DHPC [259]	Visualization of large-scale genome sequences by mapping sequences into a 2D using the space-filling function of Hilbert-Peano mapping	http://www.hpcurve.com	Standalone
DNAPlotter [260]	A Java application for generating circular and linear representations of genomes. Makes use of the Artemis libraries	http://www.sanger.ac.uk/resources/software/dnaplotter/	Standalone
FilooT [261]	A visualization tool for exploring genomic data	No URL	Standalone
GBrowsesyn [262]	GBrowse-based synteny browser designed to display multiple genomes, with a central reference species compared with two or more additional species	http://gmod.org/wiki/GBrowse_syn	Standalone
GenomeComp [263]	A tool for summarizing, parsing and visualizing the genome-wide sequence comparison results derived from voluminous BLAST textual output	http://www.mgc.ac.cn/GenomeComp/	Standalone
GenomeMatcher [264]	A dot-plot-based viewer for DNA sequence comparison	http://tinyurl.com/genomematcher/	Web-based
GenPlay Multi-Genome [265]	A tool to compare and analyze multiple human genomes in a graphical interface	http://genplay.einstein.yu.edu	Standalone
ggbio [266]	R library to visualize particular genomic regions and genome-wide overviews	http://www.bioconductor.org/packages/2.11/bioc/html/ggbio.html	Standalone
Gramene [267, 268]	A comparative genome mapping database for grasses and a community resource for *Oryza sativa*	http://ensembl.gramene.org/genome_browser/index.html	Web-based
HilbertVis [269]	Functions to visualize long vectors of integer data by means of Hilbert curves	http://www.ebi.ac.uk/huber-srv/hilbert/	Standalone
In-GAVsv [270]	Integrative genome analysis pipeline (inGAP), which uses a Bayesian principle to detect SNPs and small insertion/deletions (indels)	http://ingap.sourceforge.net/	Standalone
Meander [271]	Hilbert plots to visually discover and explore structural variations in a genome based on read-depth and pair-end information	https://sites.google.com/site/meanderviz/	Standalone
MEDEA [272]	Genomic feature densities and genome alignments of circular genomes. Comparative genomic visualization with Adobe Flash	http://www.broadinstitute.org/annotation/medea/	Web-based
MizBee [273]	A multiscale synteny browser for exploring conservation relationships in comparative genomics data. Using side-by-side linked views, it enables efficient data browsing across a range of scales, from the genome to the gene	http://www.cs.utah.edu/~miriah/mizbee	Web-based
MuSiC [274]	Identifying mutational significance in cancer genomes	http://gmt.genome.wustl.edu	Standalone
ngs.plot [275]	Quick mining and visualization of NGS data by integrating genomic databases	https://github.com/shenlab-sinai/ngsplot	Standalone
PhIGs [276]	Ideogram-style interactive display of orthologs across >75 genomes	http://phigs.org	Web-based
PSAT [277]	A web tool to compare genomic neighborhoods of multiple prokaryotic genomes	http://www.nwrce.org/psat	Web-based
Seevolution [278]	Interactive 3D environment that enables visualization of diverse genome evolution processes	http://seevolution.org	Standalone
Sybil [279]	Comparative genome data, particularly protein and gene clustered data	http://sybil.sourceforge.net/	Web-based
SynView [238]	A GBrowse-compatible approach to visualizing comparative genome data	http://gmod.org/wiki/SynView	Standalone
TREAT [280]	A bioinformatics tool for variant annotations and visualizations in targeted and exome sequencing data	http://ndc.mayo.edu/mayo/research/biostat/stand-alone-packages.cfm	Standalone
UCSC Genome Browser [281]	Conservation tracks within the popular UCSC genome browser	http://genome.ucsc.edu/cgi-bin/hgGateway/	Web-based
Vanno [282]	A visualization-aided variant annotation tool	http://cgts.cgu.edu.tw/vanno	Web-based
Variant View [283]	Features an information-dense visual encoding that provides maximal information at the overview level, in contrast to the extensive navigation required by currently prevalent genome browsers	http://www.cs.ubc.ca/labs/imager/tr/2013/VariantView/	Web-based
VISTA [108]	A comprehensive suite of programs and databases for comparative analysis of genomic sequences	http://genome.lbl.gov/vista/index.shtml	Web-based
VSV, VISTA-Dot [284, 285]	Three-scale viewer for synteny and dynamic, interactive dot plots for whole-genome DNA alignments	http://genome.jgi-psf.org/synteny/	Web-based

Although tremendous progress has been made in genomic visualization and very large amounts of money have been invested in such projects, genome browsers [110] still need to address major problems. One of the biggest challenges is the integration of data in different formats (such as genomic and clinical data) as society enters the personalized medicine era. Furthermore, navigation at different resolution or granularity levels and smooth scaling are necessary as long as simultaneous comparisons across millions of elements [111] remains a bottleneck. Newer infrastructure and software that allow on-the-fly calculations both in the front end and the back end would definitely be a step forward. Finally, similarly to network biology, time-series data visualization is one of the great challenges. For example, in a hypothetical scenario in which it is required to follow genomic rearrangements over time during tumor development, time-series data visualization would be an invaluable tool. Motion integration and visualization using additional dimensions could be possible solutions. Overall, it would be unrealistic to expect an ideal universal genome browser that serves all the possible purposes in the field.

### Visualization and analysis of expression data

**Fig. 5:**
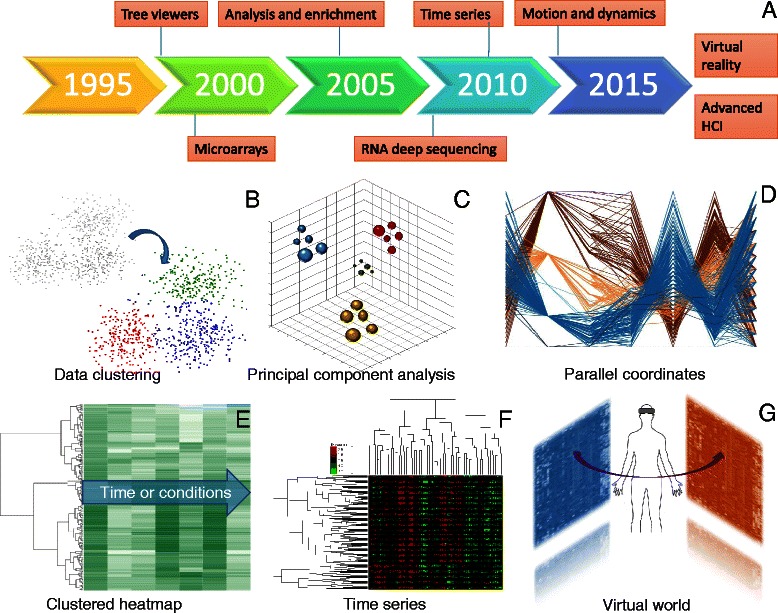
Multivariate analyses and visualization. **a** Timeline of the emergence of relevant technologies and concepts. **b** Visualization of k-means partitional clustering algorithm. **c** 3D visualization of a principal component analysis. **d** Visualization of gene-expression measures across time using parallel coordinates. **e** Visualization of gene-expression clustering across time. **f** 2D hierarchical clustering to visualize gene expressions against several time points or conditions. **g** Hypothetical integration of analyses and expression heatmaps and the control of objects by VR devices

Microarrays [112] and RNA sequencing [87] are the two main high-throughput techniques for measuring expression levels of large numbers of genes simultaneously. Both methods are revolutionary as one can simultaneously monitor the effects of certain treatments, diseases and developmental stages on gene expression across time (Fig. 5a) and for multiple transcript isoforms. Although microarrays and RNAseq technologies are comparable to each other [113], the latter tends to dominate, especially as sequencing technologies have improved, and there now are robust statistics to model the particular noise characteristics of RNAseq, particularly for low expression [114]. Microarrays are still cheaper and in some contexts may be more convenient as their analysis is still simpler and requires less computing infrastructure.

In both cases, a typical analysis procedure is first to normalize experimental and batch differences between samples and then to identify up- and downregulated genes based on a fold-change level when comparing across samples, such as between a healthy and a non-healthy tissue. Statistical approaches are used to assess how reliable fold-change measurements are for each transcript of interest by modeling variation across transcripts and experiments. Subsequently, functional enrichment is performed to identify pathways and biological processes in which the up- and downregulated genes may be involved. Although there are numerous functional enrichment suites [115], David [116], Panther [117] and WebGestalt [118] are among the most widely used.

When gene expression is measured across many time points or conditions so as to observe, for example, the expression patterns following treatment, various analyses can be taken into consideration. Principal component analysis or partitional clustering algorithms such as k-means [119] can be used to group together genes with similar behavior patterns. Scatter-plotting is the typical visualization to represent such groupings. Thus, each point on a plane represents a gene and the closer two genes appear, the more similar they are (Fig. 5b, c).

When one wants to categorize genes with similar behavior patterns across time (Fig. 5d), hierarchical clustering based on expression correlation can be performed. Average linkage, complete linkage, single linkage, neighbor joining [120] and UPGMA [121] are the most widely used methods. In such approaches, an all-against-all distance or correlation matrix that shows the similarities between each pair of genes is required and genes are placed as leaves in a tree hierarchy. The two most widely used correlation metrics for expression data are the Spearman and Pearson correlation metrics. A list of tree viewers for hierarchical clustering visualization is presented in Table 7. A more advanced visualization method is combining trees with heatmaps (Fig. 5e): genes are grouped together according to their expression patterns in a tree hierarchy and the heat map is a graphical representation of individual gene-expression values represented as colors. Darker colors indicate a higher expression value and vice versa. An even more complex visualization of a 2D hierarchical clustering is shown in Fig. 5f, in which genes are clustered based on their expression patterns across several conditions (vertical tree on the left) and conditions are clustered across genes (horizontal tree). The heatmap shows the correlations between gene groups and conditions by allowing the researcher to come to conclusions about whether a group of genes is affected by a set of conditions or not. Heatmaps do, however, have significant drawbacks with regards to color perception. Perception of the color of a cell in a heatmap is shaped by the color of the surrounding cells, so two cells with identical color can look very different depending on their position in the heatmap.

**Table 7:** Tree viewers and phylogenies

Tool and references	Description	URL
ARB [123]	A graphically oriented package comprising various tools for sequence database handling and data analysis	http://www.arb-home.de/
Bio.Phylo [286]	A unified toolkit for processing, analyzing and visualizing phylogenetic trees in Biopython	http://biopython.org
Dendroscope [125]	Software for visualizing phylogenetic trees and rooted networks	http://ab.inf.uni-tuebingen.de/software/dendroscope/
ETE Toolkit [287]	Python programming toolkit that assists in the automated manipulation, analysis and visualization of phylogenetic and other types of trees	http://etetoolkit.org/
EvolView [288]	Tool for displaying, managing and customizing phylogenetic trees	http://www.evolgenius.info/evolview.html
iTOL [126]	Online tool for the display and manipulation of phylogenetic trees	http://itol.embl.de/
MEGA [122]	Integrated tool for phylogenetic analysis and visualization	http://www.megasoftware.net/
NJplot [124]	A tree drawing program able to draw any phylogenetic tree expressed in the Newick phylogenetic tree format	http://doua.prabi.fr/software/njplot
OneZoom [289]	Committed to heightening awareness about the diversity of life on earth and its evolutionary history	http://www.onezoom.org/
Paloverde [290]	3D visualization of phylogenetic structure of moderately large trees on the scale of 100–2500 leaf nodes	http://loco.biosci.arizona.edu/paloverde/paloverde.html
PhyloDraw [291]	Drawing tool for creating phylogenetic trees	http://jade.cs.pusan.ac.kr/phylodraw/
PhyloExplorer [292]	Tool to facilitate assessment and management of phylogenetic tree collections	http://www.ncbi.orthomam.univ-montp2.fr/phyloexplorer/
PhyloWidget [293]	Program for viewing, editing and publishing phylogenetic trees online	http://www.phylowidget.org/
TreeDyn [294]	TreeDyn links unique leaf labels to lists of variables/values pairs of annotations, independently of the tree topologies	http://www.treedyn.org/
TreeGraph [295]	A graphical editor for phylogenetic trees that allows many graphical formats for the elements of the tree	http://treegraph.bioinfweb.info/
TreeQ-Vista [296]	Designed for presenting functional annotations in a phylogenetic context	http://genome.lbl.gov/vista/TreeQVista/
TreeVector [297]	Web utility to create and integrate phylogenetic trees as Scalable Vector Graphics (SVG) files	http://supfam.cs.bris.ac.uk/TreeVector/
TreeVolution [298]	Java tool to support visual analysis of phylogenetic trees	http://vis.usal.es/treevolution
T-REX [299]	Web server dedicated to the reconstruction of phylogenetic trees and reticulation networks and to the inference of horizontal gene transfer events	http://www.trex.uqam.ca/
ViPhy [300]	Comparison of multiple phylogenetic trees	http://www.gris.tu-darmstadt.de/research/vissearch/projects/ViPhy/

**Fig. 6:**
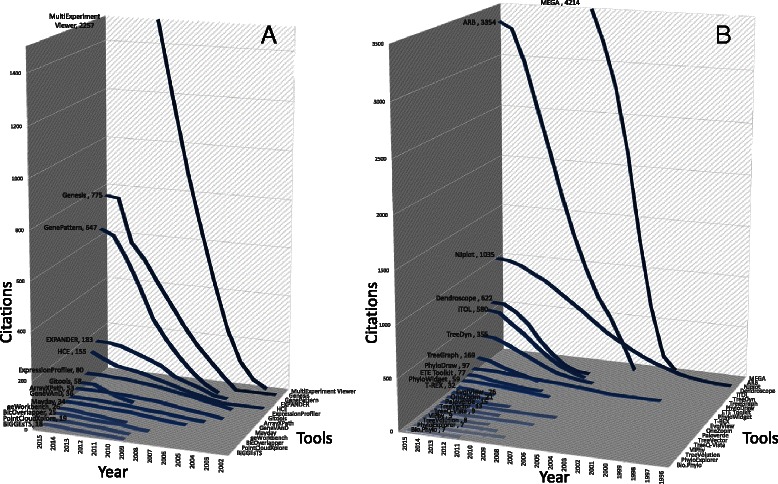
Citation trends and tools for gene-expression analysis. **a** Citations of microarray/RNAseq visualization tools based on Scopus. **b** Citations of tree viewers based on Scopus. The numbers of citations of each tool in 2015 are shown after its name

Although RNAseq analysis is still an active field, microarray analysis has matured a lot over the past 15 years and many suites for analyzing such data are currently available (Table 8). To identify the key players in the field of microarray/RNAseq visualization we followed the citation patterns of the available tools from Scopus (Fig. 6). MEGA [122], ARB [123], NJplot [124], Dendroscope [125] and iTOL [126] are the most widely used tree viewers to visualize phylogenies and hierarchical clustering results. MultiExperiment Viewer [127], Genesis [128], GenePattern [129] and EXPANDER [130] are advanced suites that can perform various multivariate analyses such as the ones discussed in this section. Nevertheless, the commercial GeneSpring platform and the entire R/BioConductor framework [131, 132] are mostly used in such analyses.

**Table 8:** Microarray and RNAseq analysis viewers

Tool and references	Description	URL
ArrayXPath [201]	Mapping and visualizing microarray gene-expression data with integrated biological pathway resources using scalable vector graphics	http://www.snubi.org/software/ArrayXPath/
BicOverlapper [301, 302]	Supports visual analysis of gene expression by means of biclustering	http://vis.usal.es/bicoverlapper/
BiGGEsTS [303]	Tool providing an integrated environment for the biclustering analysis of time-series gene-expression data	http://tinyurl.com/BiGGEsTS/
eRNA [304]	RNA data analysis tool for high-throughput RNA sequencing experiments	https://sourceforge.net/projects/erna/?source=directory
EXPANDER [130]	A Java-based tool for analysis of gene-expression and NGS data	http://acgt.cs.tau.ac.il/expander/
ExpressionProfiler [305]	Web-based platform for microarray gene-expression and other functional-genomics-related data analysis	http://www.ebi.ac.uk/expressionprofiler
GenePattern [129]	Modular analysis web platform; several visualization modules available	http://genepattern.broadinstitute.org/gp/pages/login.jsf
Genesis [128]	Java package of tools to simultaneously visualize and analyze a whole set of gene-expression experiments	http://genome.tugraz.at/genesisclient/genesisclient_description.shtml
GeneVAnD [306]	Linked heatmaps, dendrograms and 2D/3D scatter plots	http://tinyurl.com/GeneVAnD/
geWorkbench [307]	A Java-based open-source platform for integrated genomics. It allows individually developed plugins to be configured into complex bioinformatic applications. Currently more than 70 available plugins supporting the visualization and analysis	http://wiki.c2b2.columbia.edu/workbench/index.php/Home
Gitools [308]	Analysis and visualization of genomic data using interactive heatmaps	http://www.gitools.org
HCE [309]	Linked heat map, profile and scatter plots; systematic exploration	http://www.cs.umd.edu/hcil/hce/
HeatmapGenerator [310]	Create customized gene-expression heatmaps from RNAseq and microarray data	http://sourceforge.net/projects/heatmapgenerator/
HeatMapViewer [311]	Interactive display of microarray experiments or the outcome of mutational studies and the study of SNP-like sequence variants	http://dx.doi.org/10.5281/zenodo.7706
Mayday [312]	A graphical user interface that supports the development and integration of existing and new analysis methods. Many linked visualizations	http://it.informatik.uni-tuebingen.de/?page_id=248/wp/
MultiExperiment Viewer [127]	Analysis suite. Heatmaps, dendrograms, profile and scatter plots	http://www.tm4.org/
PointCloudXplore [313]	Visualization of transcription data in *Drosophila* embryos. Multiple views to ease analysis of complex gene-expression data. This type of interaction blends high-dimensional information exploration with interactive, 3D visualization	http://tinyurl.com/PointCloudXplore/
RNASeqBrowser [314]	A genome browser for simultaneous visualization of raw strand specific RNAseq reads and UCSC genome browser custom tracks	http://www.australianprostatecentre.org/research/software/rnaseqbrowser
RNAseqViewer [315]	Visualization of the various data from the RNAseq analyzing process, for single or multiple samples	http://bioinfo.au.tsinghua.edu.cn/software/RNAseqViewer/
TimeSearcher [316]	Interactive querying and exploration of time-series data	http://www.cs.umd.edu/hcil/timesearcher/
TraV [317]	Visualization and analysis of multiple transcriptome sequencing experiments	http://appmibio.uni-goettingen.de/index.php?sec=serv

Concerning the future of multivariate data visualization, new HCI techniques and VR devices could allow parallel visualizations, analyses and data integration simultaneously (Fig. 5g).

### Programming languages and complementary libraries for building visual prototypes

Although the field of biological data visualization has been active for 25 years, it is still evolving rapidly today, as the complexity and the size of results produced by high-throughput approaches increase. Although most of the current software is offered in the form of standalone distributions, a shift towards web visualization is growing. Important features of modern visualization tools include: interactivity; interoperability; efficient data exploration; quick visual data querying; smart visual adjustment for different devices with different dimensions and resolutions; fast panning; fast zooming in or out; multilayered visualization; visual comparison of data; and smart visual data filtering. As functions and libraries implementing these features for standalone applications become available, similar libraries for web visualizations immediately follow. Therefore, in this section we discuss the latest programming languages, libraries and application program interfaces (APIs) that automate and simplify many of the aforementioned features, enabling higher-quality visualization implementations. It is not in the scope of this review to extensively describe all programming language possibilities for data visualization; therefore, we focus on the five languages that are mostly used for high-throughput biological data. Nevertheless, Table 9 summarizes other languages, along with generic and language-specific libraries (for R, Perl and Python), that target specific problems and make the implementation of biological data visualization more practical.

**Table 9:** Programming languages and libraries to build visual prototype

Language/Library	Description	URL
Adobe Edge	Animated, interactive web content for projects that previously required Flash	https://creative.adobe.com/products/animate
Arbor.js	Efficient, force-directed layout algorithm plus abstractions for graph organization and screen refresh handling	http://arborjs.org/
Biojs	BioJS enables a full-featured biological workbench directly in your browser	http://biojs.net/
Bonsai.js	Lightweight graphics library with an intuitive graphics API and an SVG renderer	https://bonsaijs.org/
Chart.js	Object oriented client side graphs. Data visualization in six animated, fully customizable chart types	http://www.chartjs.org/
Cube	Time-series data, built on MongoDB, Node and D3. Real-time visualizations of aggregate metrics	https://square.github.io/cube/
Cubism	D3 plugin for visualizing time series	https://square.github.io/cubism/
Cytoscape Web	Easily embed interactive networks in your website	http://cytoscapeweb.cytoscape.org/
D4	Friendly charting domain-specific language for D3	https://github.com/heavysixer/d4
Easeljs	API to work with rich graphics and interactivity with HTML5 Canvas	http://www.createjs.com/EaselJS
Ember Charts	Ember.js and d3.js based time series, bar, pie and scatter charts that are easy to extend and modify	http://addepar.github.io/
Envision	Fast, dynamic and interactive HTML5 visualizations	http://www.humblesoftware.com/envision
Flare	Interactive data visualizations in Flash (ActionScript)	http://flare.prefuse.org/
Foamtree	Tree map visualization with innovative layout algorithms and animations such as Voronoi Treemaps	http://carrotsearch.com/foamtree-overview
Highcharts.js	HTML5/JavaScript-based line, spline, area, area-spline, column, bar, pie, scatter, angular gauges, area-range, area-spline-range, column-range, bubble, box plot, error bars, funnel, waterfall and polar charts	http://www.highcharts.com/
Infovis Toolkit	A comprehensive range of tools for creating Interactive Data Visualizations for the Web	http://philogb.github.io/jit/
Jgrapht	A free Java graph library that provides mathematical graph-theory objects and algorithms	http://jgrapht.org/
Kartograph	Kartograph is a simple and lightweight framework for creating beautiful, interactive vector maps	http://kartograph.org/
Matplotlib	A Python 2D plotting library that produces publication quality figures	http://matplotlib.org/
Miso	Interactive storytelling and data visualization content	http://misoproject.com/
Netadvantage	Charts with a range of frameworks including asp.net and Silverlight. Visualization options include bar, bubble, Gantt, line, radial, scatter, spline and doughnut charts	http://www.infragistics.com/products
Orange	Data mining through visual programming or Python scripting. Components for machine learning. Add-ons for bioinformatics and text mining. Packed with features for data analytics	http://orange.biolab.si/
Paper.js	A vector graphics scripting framework that runs on top of the HTML5 Canvas	http://paperjs.org/
Pivotviewer	A Silverlight control that makes it easier to interact with massive amounts of data on the web	http://www.microsoft.com/silverlight/pivotviewer/
Polychart.js	A JavaScript graphing library capable of producing a wide array of graphics fairly easily	http://www.polychartjs.com/
Prefuse	Java-based interactive data. Data structures for tables, graphs and trees, a host of layout and visual encoding techniques, animation, dynamic queries, integrated search and database connectivity	http://prefuse.org/
Prefuse Flare	Visualization and animation for ActionScript. From basic charts and graphs to complex interactive graphics. Data management, visual encoding, animation and interaction techniques	http://flare.prefuse.org/
Ractive.js	It transforms templates into blueprints for apps that are interactive by default. Two-way binding, animations, SVG support and more	http://www.ractivejs.org/
Raphael.js	JavaScript library for vector graphics on the web. To create a specific chart or image. Crop and rotate widget	http://raphaeljs.com/
Rcharts	R package to create, customize and publish interactive JavaScript visualizations from R using a familiar lattice style plotting interface	http://rcharts.io/
Seaborn	A Python visualization library based on matplotlib. It provides a high-level interface for drawing attractive statistical graphics	http://stanford.edu/~mwaskom/software/seaborn/
Shiny	A web application framework for R to turn an analysis into interactive web applications. No HTML, CSS or JavaScript knowledge required	http://shiny.rstudio.com/
Sigma.js	A JavaScript library dedicated to graph drawing. It makes easy to publish networks on Web pages and allows developers to integrate network exploration in rich web applications	http://sigmajs.org/
Three.js	A lightweight cross-browser JavaScript library/API used to create and display animated 3D computer graphics on a web browser that supports WebGL	http://threejs.org/
Timeline.js	Visually rich interactive timelines, available in 40 languages	http://timeline.knightlab.com/
Variance	Build powerful data visualizations for the web without writing JavaScript. Wide range of visualizations	https://variancecharts.com/
Vega	A visualization grammar, a declarative format for creating, saving and sharing visualization designs. Data visualizations in JSON format and interactive views using either HTML5 Canvas or SVG	http://trifacta.github.io/vega/
Vida.io	A way to build reusable cloud visualizations: clone visualization templates, customize without coding skills and embed or share in the cloud	https://vida.io/
Vis	A data visualization platform designed to assist investigative journalists, activists and others in mapping complex business or crime networks	http://vis.occrp.org/
Visual Sedimentation	A JavaScript library for visualizing streaming data, inspired by the process of physical sedimentation. jQuery (to facilitate HTML and JavaScript development) and Box2DWeb (for physical world simulation)	http://www.visualsedimentation.org/
WebGL	A JavaScript API for rendering interactive 3D computer graphics and 2D graphics within any compatible web browser without the use of plugins	https://www.khronos.org/webgl/

#### Processing

‘Processing’ is a programming language and a development platform for writing generative, interactive and animated standalone applications. Basic shapes such as lines, triangles, rectangles and ellipses, inner/outer coloring and basic operations such as transformations, translations, scaling and rotations can be implemented in a single line of code and each shape can be drawn within a canvas of a given dimension and a given refresh rate. It is designed for easier implementations of 2D dynamic visualizations but it also supports 3D rendering, although not optimized. Its core library is now extended by more than 100 other libraries and it is one of the best documented languages in the field. The integrated development environment allows exporting of executable files for all Windows, MacOS and Linux operating systems as well as Java applet .jar files. Finally, it can be used as an excellent educational tool for computer programming fundamentals in a visual context. It is free for download, can easily be plugged in a Java standalone application, and is fully cooperative with the NetBeans and Eclipse environments. Code examples and tutorials can be found at [133].

#### Processing.js

Java applets were an easy way to run standalone applications within web browsers. This technology has now mainly been abandoned because of security considerations. To avoid JavaScript’s complexity and compensate for applet limitations, Processing.js was implemented, as the sister project of the popular Processing programming language, to allow interactive web visualization. It is a mediator between HTML5 and Processing and is designed to allow visual prototypes, digital arts, interactive animations, educational graphs and so on to run immediately within any HTML5-compatible browser, such as Firefox, Safari, Chrome, Opera or Internet Explorer. No plugins are required and one can code any visualization directly in the Processing language, include it in a web page, and let Processing.js bridge the two technologies. Processing.js brings the best of visual programming to the web, both for Processing and web developers. Code examples and tutorials can be found at [134].

#### D3

D3 is the main competitor of Processing/Processing.js and has gained ground over recent years. It was initially used to generate scalable vector graphics (SVG). Like Processing.js, it is designed for powerful interactive web visualizations and it comes with its own JavaScript-like syntax. It is a JavaScript library for manipulating document object model objects and a programming interface for HTML, XML and SVG. The idea behind this approach is to load data into a browser and then generate document object model elements based on that data. Subsequently, one can apply data-driven transformations on the document. This avoids proprietary representation and affords extraordinary flexibility. With minimal overhead, D3 is extremely fast and supports large datasets and dynamic behaviors for interaction and animation. D3’s functional style allows code reuse through a diverse collection of components and plugins. It is extensively documented and code examples can be found at [135].

#### Flash

Adobe Flash was once the industry standard for authoring innovative, interactive content. In conjunction with the platform’s programming language, ActionScript, Flash allows designers to implement dynamic visualization, opening up many possibilities for creativity. Some of the most pioneering, best practice visualizations built in Flash can be found with online news and media sites, introducing interactivity to supplement and enhance the presentation of information. Because of the lack of support for Flash across Apple’s suite of devices and the emergence of competing developments, demanding less computational power, including D3 and HTML5, this technology is now fading.

#### Java3D

Java 3D is an API, acting as a mediator between OpenGL and Java and enables the creation of standalone 3D graphics applications and internet-based 3D applets. It is easy to use and provides high-level functions for creating and manipulating 3D objects in space and their geometry. Programmers initially create a virtual world and then place any 3D object anywhere in this world. Rotation in three axes, zooming in or out and translation of the whole canvas are functions are offered by default, and the hierarchy of the transformation groups define the 3D transformations that can be applied individually to an object or a set of objects. Java3D code can be compiled under any of the Windows, MacOS and Unix Systems.

## Conclusion

### The future of biological data visualization

Biological data visualization is a rapidly evolving field. Nevertheless, it is still in its infancy. Hardware acceleration, standardized exchangeable file formats, dimensionality reduction, visual feature selection, multivariate data analyses, interoperability, 3D rendering and visualization of complex data at different resolutions are areas in which great progress has been achieved. Additionally, image processing combined with artificial-intelligence-based pattern recognition, new libraries and programming languages for web visualization, interactivity, visual analytics and visual data retrieval, storing and filtering are still ongoing efforts with remarkable advances over the past years [58, 136, 137]. Today, many of the current visualization tools serve as front ends for very advanced infrastructures dedicated to data manipulation and have driven significant advances in user interfaces. Although the implementation of sophisticated graphical user interfaces is necessary, the effort to minimize back-end calculations is of great importance. Unfortunately, only a limited number of visualization tools today take advantage of libraries designed for parallelization. Multi-threading, for example, allows the distribution of computational tasks in terminals over the network, and CUDA (available on Nvidia graphic cards) allows parallel calculations at multiple graphical processing units.

Despite the fact that multiple screens, light and laser projectors and other technologies partially solve the space limitation problem, HCI techniques are changing the rules of the game and biological data visualization is expected to adjust to these trends in the longer term. 3D control can be achieved without intermediate devices such as mouse, keyboards or touch screens [138] in modern perceptual input systems. Sony’s EyeToy, Playstation Eye and Artag, for example, use non-spatial computer vision to determine hand gestures. Similarly, the Nintendo Wii and Sony Move devices support object manipulation in 3D space. These actions are mediated through the detection of the position in space of physical devices held by the user or, even more impressively, through immediate tracking of the human body or parts of the human body. Equally impressive is the prospect of ocular tracking, one implementation of which has recently been introduced by the VR startup Fove. The Fove headset tracks eye movement and translates into spatial movement or even other types of action within the simulated 3D space. The recently implemented Molecular Control Toolkit [139] is a characteristic example of a new API based on the Kinect and Leap Motion devices (which track the human body and human fingers, respectively) to control molecular graphics such as 3D protein structures. Moreover, large screens, tiled arrays or VR environments should be taken into consideration by programmers and designers as they become more and more affordable over time. A great benefit of such technologies is that they allow the representation of complete datasets without the need for algorithms dedicated to dimensionality reduction, which might lead to information loss.

VR environments are expected to bring a revolution in biological data visualization as one could integrate metabolomics networks and gene expression in virtual worlds, as in MetNet3D [140], or create virtual universes of living systems such as a whole cell [59, 141–144]. A visual representation of the whole cell with its components in an immense environment in which users can visually explore the location of molecules and their interaction in space and time could lead to a better understanding of the biological systems. Oculus Rift (which promoted the reemergence of VR devices), Project Morpheus, Google Cardboard, Sony Smart Eyeglass, HTC Vive, Samsung Gear VR, Avegant Glyph, Razer OSVR, Archos VR Headset and Carl Zeiss VR One are state-of-the-art commercial devices that offer VR experiences. All of them overlay the user’s eyesight with some kind of screen and aim to replace the field of view with a digital 3D alternative. Between them, those devices use many technologies and new ideas such as the monitoring of the position of the head (allowing for more axes of movement), the substitution of the VR screen with smartphones (thus harnessing efficient modern smartphone processors), eye tracking and projection of images straight onto the retina.

Approaching the problem from a different angle, Google Glass, HoloLens and Magic Leap offer an augmented reality experience (the latter is rumored to achieve that by projecting a digital light field into the user’s eye). Augmented reality can facilitate the learning process of the biological systems because it builds on exploratory learning. This allows scientists to visualize existing knowledge, whereas the unstructured nature of augmented reality could allow them to construct knowledge themselves by making connections between information and their own experiences or intuition and thus offer novel insights to the studied biological system [145]. Efforts such as the Visible Cell [141] and CELLmicrocosmos have already begun. The Visible Cell project aims to inform advanced *in silico* studies of cell and molecular organization in 3D using the mammalian cell as a unitary example of an ordered complex system; the CELLmicrocosmos integrative cell modeling and stereoscopic 3D visualization project is a typical example of the use of 3D vision.

Finally, starting from a living entity, the process of digitizing it, visualizing it, placing it in virtual worlds or even recreating it as a physical object using 3D printing is no longer the realm of science fiction. Data visualization and biological data visualization are rapidly developing in parallel with advances in the gaming industry and HCI. These efforts are complementary and there are already strong interactions developing between these fields, something that is expected to become more obvious in the future.
